# Clinical Outcomes Associated with Statin Use in Pulmonary Embolism: A Systematic Review of Observational Studies

**DOI:** 10.3390/jcm14238333

**Published:** 2025-11-24

**Authors:** Walaa A. Alshahrani, Abdulmajeed M. Alshehri, Majed S. Al Yami

**Affiliations:** 1 King Abdullah International Medical Research Center (KAIMRC), Riyadh 11481, Saudi Arabia; alshahraniiwa@gmail.com (W.A.A.); shehriabdul@ksau-hs.edu.sa (A.M.A.); 2 Department of Pharmacy Practice, College of Pharmacy, King Saud bin Abdulaziz University for Health Sciences, Riyadh 11481, Saudi Arabia; 3 King Abdulaziz Medical City, National Guard Health Affairs, Riyadh 14611, Saudi Arabia

**Keywords:** pulmonary embolism, statins, venous thromboembolism, mortality

## Abstract

**Background**: Pulmonary embolism (PE) represents one of the most serious complications of venous thromboembolism (VTE). Conflicting studies indicate that statins could play a role in preventing recurrent VTE or improving survival in patients with PE. This review aims to assess the impact of statin use on clinical outcomes in patients with PE. **Methods**: A systematic search was conducted in PubMed and the Cochrane Library to identify studies evaluating the impact of statin therapy in patients with PE. The search covered all records from inception to 21 September 2025. Studies were deemed eligible if they included adult patients with PE, investigated statin and reported clinical outcomes such as mortality, recurrent PE, recurrent VTE, or deep vein thrombosis (DVT). The methodological quality of the included observational studies was assessed using the Newcastle–Ottawa Scale (NOS). **Results**: A total of twelve observational studies were included; sample sizes ranged from fewer than 400 patients to nearly 200,000, with follow-up durations spanning from 30 days to more than 5 years. Statin use was associated with reduced short-term and all-cause mortality in large registry analyses, though smaller studies showed no difference. Most cohorts reported lower VTE recurrence among statin users, with some variation across settings. Overall, evidence suggests a potential benefit of statins in reducing mortality and VTE recurrence in patients with pulmonary embolism. **Conclusions**: Real-world data suggest that statin therapy may reduce both short-term mortality and VTE recurrence following PE, though findings vary depending on study quality and population characteristics. Larger prospective studies are required to confirm these findings.

## 1. Introduction

Pulmonary embolism (PE) represents one of the most serious complications of venous thromboembolism (VTE). It continues to be a major contributor to morbidity and mortality worldwide despite major progress in imaging, diagnosis, and anticoagulant therapy [1,2]. Even with effective treatment, some patients experience recurrence or develop chronic complications such as chronic thromboembolic pulmonary hypertension, which still limits long-term outcomes [3,4]. These challenges have prompted growing interest in additional or supportive therapies that could enhance recovery and reduce late events beyond routine anticoagulation.

Statins—hydroxymethylglutaryl-CoA (HMG-CoA) reductase inhibitors—are among the most commonly prescribed cardiovascular drugs. Their lipid-lowering effect is well documented, but accumulating evidence suggests broader “pleiotropic” actions, including anti-inflammatory, antithrombotic, and endothelial-protective properties [5,6,7,8]. Experimental and translational studies indicate that statins can dampen tissue-factor expression, enhance nitric-oxide availability, and stabilize the vascular endothelium—mechanisms that might, at least theoretically, influence thrombosis risk [8,9]. This biological background provides a rationale for exploring whether statins could play a role in preventing recurrent VTE or improving survival in patients with PE.

Evidence so far comes mostly from observational research, and findings have not always aligned. Large randomized studies such as the JUPITER trial showed fewer first-time VTE events among people taking rosuvastatin [10], yet similar randomized data in patients who already have PE are missing. Observational cohorts and registry analyses have suggested possible reductions in mortality and VTE recurrence, but these results vary depending on population, study design, and how thoroughly confounding factors were handled [11,12].

Because of these discrepancies, a systematic evaluation of real-world data is needed to better understand the relationship between statin therapy and patient outcomes after PE. The present review therefore compiles and critically appraises observational studies assessing how statin use relates to mortality and VTE recurrence in individuals diagnosed with PE.

## 2. Materials and Methods

### 2.1. Search Strategy, Study Selection, and Data Extraction

A systematic search was conducted in PubMed and the Cochrane Library to identify studies evaluating the impact of statin therapy in patients with pulmonary embolism (PE). The PubMed search strategy combined the terms (“Hydroxymethylglutaryl-CoA Reductase Inhibitors” [MeSH Terms] OR statins OR atorvastatin OR simvastatin OR rosuvastatin OR pravastatin OR lovastatin OR fluvastatin OR pitavastatin) AND (“Pulmonary Embolism” [MeSH Terms] OR pulmonary embolism). A similar strategy was applied to the Cochrane Library using equivalent keywords. The electronic search covered all records from inception to 21 September 2025. Studies were deemed eligible if they included adult patients with PE, investigated statin and reported clinical outcomes such as mortality, recurrent PE, recurrent VTE, or deep vein thrombosis (DVT). We included observational studies such as cohort, case–control, prospective, and retrospective designs. Exclusion criteria were applied to studies that were secondary evidence (systematic reviews, meta-analyses, or guidelines), studies with non-eligible designs (case reports, animal experiments, or genetic instruments), and studies that were out of scope due to population age, lack of statin exposure, or outcomes not specifically related to PE.

### 2.2. Quality Assessment and Risk of Bias

The methodological quality of the included observational studies was assessed using the Newcastle–Ottawa Scale (NOS). This tool evaluates three domains: Selection (0–4 stars), Comparability (0–2 stars), and Outcome (0–3 stars), with a maximum score of 9. Studies scoring 7–9 stars were considered high quality, 5–6 stars fair quality, and ≤4 stars low quality. Two reviewers independently performed the assessments, and disagreements were resolved through discussion. This systematic review reported according to the Preferred Reporting Items for Systematic Reviews and Meta-Analyses (PRISMA) statement [13]. The study was registered in the International Prospective Register of Systematic Reviews (PROSPERO) with the registration number CRD420251166536.

### 2.3. Data Synthesis and Analysis

Conducting a meta-analysis was not feasible due to the observed variability among the studies. Effect estimates (hazard ratios, odds ratios, or adjusted hazard ratios) were extracted and presented as reported in the original publications. No statistical conversion or standardization was performed due to differences in study design, outcome definitions, and follow-up duration. Instead, the findings of the included studies were synthesized in a systematic approach. The results were systematically presented according to the study designs, and the interventions evaluated, highlighting the outcomes and conclusions of each study. It is important to note that although both Siniscalchi et al. (2022) and Siniscalchi et al. (2024) [14,15] utilized data from the RIETE registry, they examined distinct cohorts: the former included patients with acute pulmonary embolism, whereas the latter included only those with isolated deep vein thrombosis, excluding any with PE. Therefore, there was no overlap in the patient populations.

## 3. Results

### 3.1. Search Results and Study Characteristics

**Figure 1:**
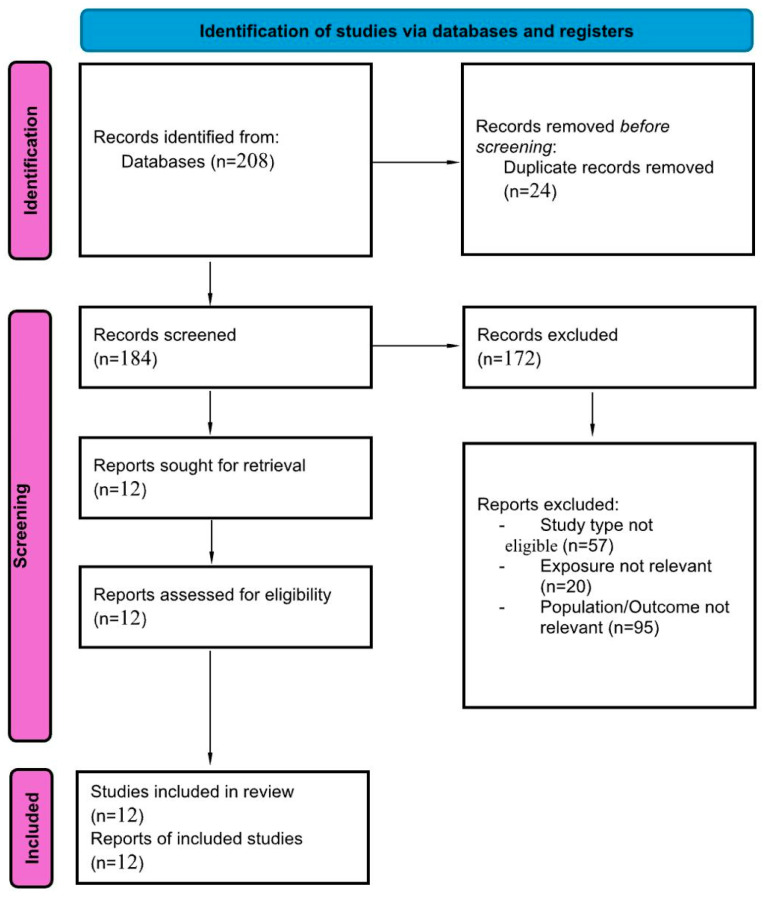
PRISMA flow diagram for selection of studies for the systematic review.

**Figure 2:**
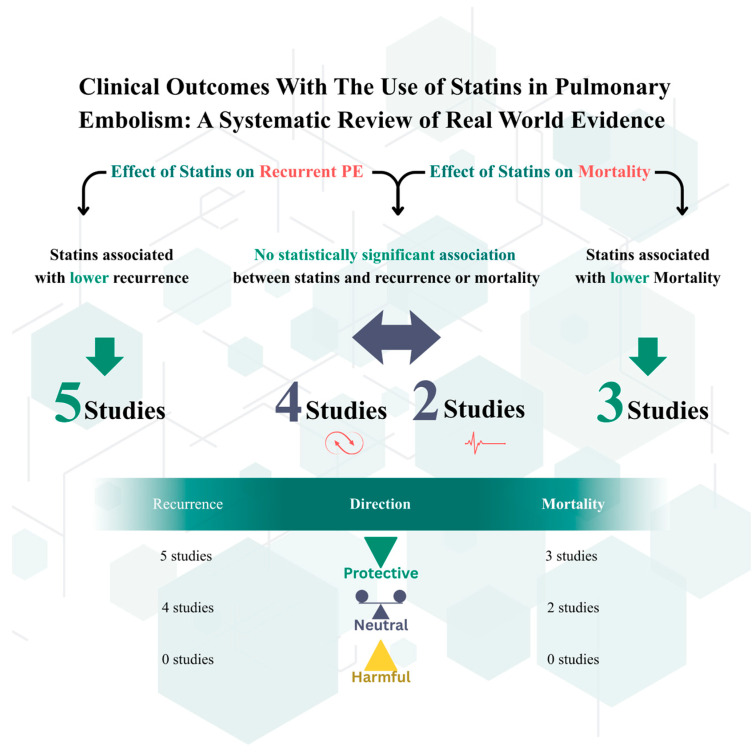
Visual abstract summarizing clinical outcomes with statin therapy in pulmonary embolism.

The literature yielded 159 records from PubMed and 47 from the Cochrane Library, for a total of 206 records. In addition, two eligible studies were identified through manual searching of reference lists, giving a combined total of 208 records. After removal of 24 duplicates (including three triple duplicates), 184 unique records were available for screening. Overall, twelve observational studies were included, comprising large national cohorts from Denmark and the Netherlands, multicenter international registries (RIETE), population-based datasets from the United States, and smaller single-center analyses from Austria and China (Figure 1). Sample sizes ranged from fewer than 400 patients to nearly 200,000, with follow-up durations spanning from 30 days to more than 5 years as shown in Table 1. Across studies, statin users were generally older and had more comorbidities than non-users. According to the NOS, the overall quality of included studies was mostly good, with four large nationwide cohorts (Nguyen, Schmidt, Smith, Biere-Rafi) rated as good to high quality (7–9 stars). More recent retrospective analyses and registry-based studies (e.g., Wang, Siniscalchi, Gressenberger) were generally rated as fair (5–6 stars), primarily due to residual confounding and limited adjustment. The ecological WHO mortality study was also rated as fair given the absence of patient-level data. A detailed breakdown is provided in Table 2. Additionally, Figure 2 provides a visual abstract summarizing the overall findings of the included studies, illustrating the direction and strength of association between statin therapy and key clinical outcomes (recurrent PE and mortality).

### 3.2. Summary of the Included Studies

In a Dutch population-based cohort using the PHARMO Record Linkage System, Biere-Rafi and colleagues evaluated whether statin therapy reduces the risk of recurrent pulmonary embolism (PE). The study included patients hospitalized for acute PE between 1998 and 2008, with follow-up data on prescriptions for statins and vitamin K antagonists. Using Cox regression analyses with statin exposure as a time-varying covariate, the investigators found that statin use was associated with a significantly reduced risk of recurrent PE (adjusted HR 0.50, 95% CI 0.36–0.70), regardless of ongoing anticoagulation. A dose–response relationship was observed, with greater benefit among those using potent statins. Statin therapy also conferred lower risks of cardiovascular events and all-cause mortality, suggesting a potential role as an adjunct or alternative in long-term secondary prevention of PE [16].

Nguyen and colleagues conducted a large nationwide Danish cohort study including 44,330 patients hospitalized with venous thromboembolism (VTE) between 1997 and 2009. For pulmonary embolism (PE) specifically, statin users had an incidence rate of 12.3 per 1000 person-years compared with 19.6 per 1000 person-years among non-users, corresponding to an adjusted hazard ratio (HR) of 0.87 (95% CI 0.78–0.97), indicating a 13% reduction in recurrent PE risk. Overall, recurrence of hospitalized VTE events was substantially lower among statin users (24.4 per 1000 person-years) compared with non-users (48.5 per 1000 person-years). Adjusted analyses showed that statin therapy was associated with a 26% lower risk of recurrent VTE (HR 0.74, 95% CI 0.68–0.80). Importantly, the protective effect was most evident in patients aged ≤ 80 years, while in those older than 80, statin use was paradoxically associated with higher recurrence risk. These findings highlight an age-dependent effect of statin therapy on VTE recurrence [17].

Using Danish nationwide health registries, Schmidt and colleagues examined the association between statin therapy and recurrent venous thromboembolism (VTE) in 27,862 patients with a first-time VTE between 2004 and 2012. For pulmonary embolism (PE) specifically, current statin use was associated with a lower recurrence rate (11.9 vs. 12.4 per 1000 person-years), corresponding to an adjusted hazard ratio (HR) of 0.82 (95% CI 0.62–1.09). In time-varying analyses, the association was stronger, with an adjusted HR of 0.68 (95% CI 0.51–0.89), indicating a 32% reduction in recurrent PE risk. Statin use was categorized as current, former, or non-use, and both time-varying Cox regression and a nested case–control analysis were performed. Current statin use was associated with a lower risk of recurrence compared with non-use (adjusted HR 0.72, 95% CI 0.59–0.88), with a stronger effect observed for high-potency statins (HR 0.40, 95% CI 0.21–0.78). The findings were consistent across sensitivity analyses, including the nested case–control approach (IRR 0.55, 95% CI 0.45–0.67). The protective effect appeared particularly pronounced for recurrent deep vein thrombosis (DVT). These results suggest that statins may provide meaningful benefit in preventing recurrent VTE [18].

Smith and colleagues examined the association between statin therapy and recurrent venous thromboembolism (VTE) in a U.S.-based observational inception cohort of 2798 adults with incident VTE identified between 2002 and 2010. Among these, approximately half (53%) had pulmonary embolism (PE) as their index event, and 39% of all recurrences involved PE. Current statin use was associated with a lower rate of recurrent PE (3.9 vs. 5.2 per 100 person-years), corresponding to an adjusted hazard ratio (HR) of 0.74 (95% CI 0.59–0.94), indicating a 26% reduction in PE recurrence risk. Over the course of follow-up, 457 patients (16%) developed a recurrent event. Time-to-event models accounting for time-varying statin use demonstrated that current statin therapy was associated with a 26% lower risk of recurrence (HR 0.74, 95% CI 0.59–0.94). Among patients without clinical cardiovascular disease at baseline, the protective effect was even stronger (HR 0.62, 95% CI 0.45–0.85). The study supports the hypothesis that statins may offer secondary preventive benefits for VTE recurrence beyond lipid lowering [19].

Brækkan and colleagues investigated the association between statin therapy and recurrence of venous thrombosis in the MEGA follow-up study. Among patients with an initial pulmonary embolism (PE), incident statin use was not associated with a reduction in recurrent PE risk (adjusted HR 1.00, 95% CI 0.50–1.98), suggesting no apparent protective effect for PE specifically. The cohort comprised 2547 patients with a first venous thromboembolism (VTE), linked to pharmacy records to determine statin use during a median follow-up of 5.7 years. Using time-dependent Cox models, the authors reported that incident statin use was associated with a non-significant 22% reduction in recurrent events overall (HR 0.78, 95% CI 0.46–1.31). Subgroup analyses suggested no protective effect in patients with unprovoked first events, although statistical power was limited. Overall, the results indicated a possible modest reduction in recurrence risk, but causality remained uncertain due to the observational design [20].

Hsu and colleagues conducted a retrospective cohort study to examine whether statin use was associated with clinical outcomes in patients hospitalized with acute pulmonary embolism (PE). Among 3097 patients with confirmed PE, 522 (16.9%) were on statin therapy prior to admission. In-hospital mortality was significantly lower in statin users compared with non-users (4.0% vs. 8.4%), corresponding to an adjusted odds ratio (OR) of 0.55 (95% CI 0.34–0.88). Additionally, statin use was associated with reduced odds of developing right ventricular dysfunction (OR 0.64, 95% CI 0.42–0.97) and lower composite adverse outcomes (OR 0.67, 95% CI 0.49–0.93). After adjusting for age, comorbidities, and concurrent cardiovascular risk factors, statin therapy did not significantly affect long-term mortality or major complications, although benefits appeared greater among patients without active cancer. The findings suggest that while statins may confer short-term protective effects during acute PE hospitalization, their impact on long-term outcomes remains uncertain and warrants prospective evaluation [21].

In a large retrospective analysis of statewide health records from Indiana (2004–2017), Stewart et al. [22] assessed the effect of statin therapy on recurrence of VTE. The study included 192,908 patients diagnosed with DVT or PE, of whom 13.5% had a statin prescription. Using propensity score matching and logistic regression, the investigators demonstrated that VTE recurrence was significantly less common among statin users (16%) compared with non-users (20%). Statin therapy was associated with an adjusted odds ratio of 0.75 (95% CI 0.72–0.79), corresponding to a 25% reduction in recurrence risk. These findings support the potential role of statins as adjunctive therapy in the prevention of recurrent VTE at the population level [22].

Wang et al. [23] examined the relationship between statin therapy and recurrence of pulmonary embolism (PE) in a real-world Chinese cohort. The retrospective study included 365 patients with ICD-confirmed PE between 2017 and 2019, of whom 96 received statins and 269 did not. Over a median follow-up of 19.2 months, 15.1% of patients experienced recurrence. Using propensity score matching and multivariable logistic regression, the authors found no significant association between statin use and recurrent PE (OR 0.489, 95% CI 0.190–1.258, *p* = 0.138). The recurrence rate was comparable between statin users and non-users (15.6% vs. 14.9%). Although anticoagulant therapy was protective, and COPD and atrial fibrillation emerged as risk factors, statin therapy showed no independent benefit. Overall, the findings did not support a role for statins in preventing PE recurrence, suggesting that anticoagulation remains the only proven preventive strategy [23].

Siniscalchi et al. [14] analyzed data from the RIETE registry to determine whether statin use affects short-term mortality in patients with acute symptomatic pulmonary embolism (PE). Between 2009 and 2021, only patients with objectively confirmed PE were included, while those with isolated deep vein thrombosis (DVT) were excluded. A total of 31,169 patients were enrolled, of whom 18% were on statin therapy at baseline. Statin users tended to be older and have more comorbidities. Nevertheless, after multivariable and propensity score–matched analyses, statin therapy was associated with significantly reduced 30-day all-cause mortality (adjusted OR 0.65, 95% CI 0.56–0.76) and fatal PE (adjusted OR 0.42, 95% CI 0.28–0.62), with stronger protective effects observed at higher statin intensities. The benefit did not extend to unrelated outcomes such as cancer-related mortality, supporting biological plausibility. These results suggest that statins may improve early survival in acute PE [14].

In a subsequent analysis of the RIETE registry, Siniscalchi and colleagues examined the impact of statin therapy on mortality among patients with isolated DVT. The study included 46,440 patients enrolled between 2009 and 2022. Statin users, who were generally older and had more comorbidities, demonstrated significantly lower 3-month mortality compared with non-users (adjusted HR 0.77, 95% CI 0.69–0.86). Protective effects were consistent across different anatomical subtypes of DVT and were particularly strong among users of simvastatin, atorvastatin, and rosuvastatin. Importantly, no association was observed with major bleeding, used as a falsification endpoint. These findings suggest that statins may confer a survival benefit in patients with DVT beyond their cardiovascular effects [15].

Hagiya and colleagues performed an epidemiological analysis of pulmonary embolism–related mortality using data from the World Health Organization mortality database between 2001 and 2023. Data from 73–75 countries were included, representing more than 1.5 million participants. Global age-standardized mortality rates for PE declined from 3.49 per 100,000 in 2001 to 2.42 per 100,000 in 2023. Substantial reductions were observed in high-income regions such as Western Europe, while rates remained persistently high in Africa and showed increasing trends in lower-middle-income countries. The study underscores global disparities in PE-related mortality and highlights the need for targeted healthcare policies to reduce the burden of disease in vulnerable populations [24].

Gressenberger and colleagues performed a retrospective cohort study at the University Hospital Graz, Austria, to evaluate the effect of statin use on the severity of acute PE. The analysis included 1590 patients diagnosed between 2010 and 2019, of whom 235 (14.8%) were on statin therapy. PE severity was classified according to the 2019 European Society of Cardiology risk stratification guidelines, incorporating clinical presentation, biomarkers, and imaging of right ventricular dysfunction. Statin users were significantly older, had higher BMI, and more comorbidities compared with non-users. Initially, a smaller proportion of statin users presented with low-risk PE (12.3% vs. 19.9%), but after adjusting for confounders and performing matched analyses, statin use was not independently associated with PE severity. Moreover, no significant differences in 30-day or 2-year mortality were observed between groups. These findings indicate that statin therapy does not meaningfully alter PE severity or survival, and baseline differences in age and comorbidity likely explain the observed crude differences [25].

### 3.3. Outcomes

With respect to mortality outcomes, nine studies reported 30-day or short-term mortality. Two large registry analyses (RIETE) demonstrated significant reductions in all-cause mortality among statin users (adjusted ORs ranging from 0.65 to 0.77), with additional benefit observed for fatal PE (OR 0.42, 95% CI 0.28–0.62). By contrast, a smaller Austrian cohort and a U.S. single-center study found no significant mortality difference, and one Chinese cohort similarly reported null results.

For recurrent venous thromboembolism (VTE), eleven studies assessed outcomes, though definitions varied. Danish and Dutch cohort studies showed consistent reductions in recurrence with statin use (HRs ranging from 0.63 to 0.78), and a U.S. statewide cohort confirmed this association (adjusted OR 0.66, 95% CI 0.64–0.69; OR 0.75, 95% CI 0.72–0.79 after propensity matching). Conversely, two studies (Austria and China) found no significant association between statins and VTE recurrence during follow-up.

Pulmonary embolism (PE)–specific recurrence was reported in twelve studies. Biere-Rafi et al. (2013) [16] observed a strong protective effect (aHR 0.50, 95% CI 0.36–0.70), whereas other cohorts demonstrated more modest associations (HRs 0.82–0.91) or no clear effect. Similarly, deep vein thrombosis (DVT) recurrence was evaluated in eleven studies, with two reporting a reduced risk among statin users, while the remainder did not detect significant differences.

Overall, the evidence across heterogeneous settings suggests that statin use may confer reductions in both short-term mortality and recurrence of VTE, although null and inconsistent findings were also reported, particularly in smaller cohorts as shown in Table 3.

## 4. Discussion

The findings of this systematic review suggest that statin therapy may have a beneficial impact on outcomes among patients with pulmonary embolism (PE). Across the included observational studies, statin use was consistently associated with lower rates of recurrent venous thromboembolism (VTE) and pulmonary embolism recurrence, as well as reduced short-term mortality. These benefits appeared more pronounced in large nationwide cohorts and international registries, whereas smaller, single-center studies yielded mixed or neutral findings. Overall, the pattern of evidence indicates that the pleiotropic anti-inflammatory and endothelial-stabilizing effects of statins may contribute to improved vascular stability and reduced thrombotic recurrence beyond their lipid-lowering role.

An important methodological aspect that may contribute to variability among studies is the way statin exposure was modeled. Some cohorts, such as Wang et al. [23] treated statin use as a binary variable, while others, including Nguyen and Schmidt, used time-varying exposure models. Time-varying approaches better account for changes in treatment status over time and reduce immortal-time bias, potentially providing more accurate risk estimates. These methodological differences may partly explain the heterogeneity observed across studies. Additionally, differences in the type of reported effect estimates (HRs, ORs, and aHRs) may limit direct numerical comparison across studies; therefore, our synthesis focused on the overall direction and consistency of associations rather than pooled magnitude. Although both the 2022 and 2024 analyses were derived from the RIETE registry, they evaluated completely distinct cohorts: the 2022 analysis included only patients with acute symptomatic pulmonary embolism, whereas the 2024 analysis included only isolated deep vein thrombosis and specifically excluded any PE cases. Therefore, there was no overlap in patient populations and no double-counting in the synthesis.

Although JUPITER and HOPE-3 do not evaluate statin therapy for secondary prevention after PE, they are referenced here solely to support the biological plausibility of statin-mediated antithrombotic and anti-inflammatory effects, rather than as direct clinical evidence for post-PE management. When compared with randomized controlled trials, the findings of this review are broadly consistent in biological direction but differ substantially in population and clinical context. Evidence so far, however, comes mostly from observational research, and findings have not always aligned. Large randomized studies such as the JUPITER trial showed fewer first-time VTE events among individuals taking rosuvastatin [10], reporting a 43% reduction in risk (HR 0.57, 95% CI 0.37–0.86). Similarly, the HOPE-3 trial demonstrated a 36% reduction in VTE risk (HR 0.64, 95% CI 0.42–0.96) among participants receiving rosuvastatin compared with placebo [26]. However, both trials evaluated statins primarily for primary prevention of cardiovascular disease and VTE in generally healthy or at-risk individuals, not for secondary prevention after established pulmonary embolism. In contrast, real-world observational evidence from large national registries and retrospective cohorts—including those by Nguyen (2013), Schmidt (2014), and Siniscalchi (2022)—has consistently shown lower recurrence rates and mortality among statin users [14,17,18]. Together, these findings suggest that while randomized data confirm a biologically plausible protective effect of statins against first-time VTE, real-world studies extend this potential benefit to secondary outcomes following PE.

This review’s strengths include its comprehensive inclusion of diverse real-world cohorts across multiple healthcare systems and its rigorous quality assessment using the Newcastle–Ottawa Scale. Together, these strengthen external validity and represent the largest synthesis of observational data on this topic to date. However, several limitations must be acknowledged: most included studies were retrospective, introducing possible confounding and treatment selection bias; outcome definitions and follow-up durations varied widely; and statin exposure was often assessed from prescription data rather than verified adherence. Additionally, the Hsu et al. (2021) [21] study did not provide a clearly stratified count of PE cases in the primary publication; only aggregated population data were available. As a result, PE case numbers used in this review were extracted from the overall cohort description, which limits interpretability of PE-specific outcomes and introduces additional uncertainty. In addition, most studies did not report thrombophilia status (e.g., Factor V Leiden, protein C/S deficiency), despite its known influence on VTE recurrence risk. This absence limits our ability to differentiate whether the potential protective effects of statins vary between provoked and unprovoked or thrombophilia-associated VTE events. Furthermore, the duration and intensity of anticoagulation therapy (OAC/NOAC) were inconsistently reported across the included studies. This prevents assessment of whether shorter or incomplete anticoagulation contributed to VTE or PE recurrence, representing an additional source of confounding. Also the lack of randomized confirmation limits the ability to establish causality. Finally, other databases such as Embase, Web of Science, and Scopus were not included, which may have limited the comprehensiveness of the search. Future prospective and randomized investigations are warranted to clarify the specific role of statins in PE management and confirm their impact on mortality and recurrence outcomes.

## 5. Conclusions

Overall, real-world data suggest that statin therapy may reduce both short-term mortality and VTE recurrence following pulmonary embolism, though findings vary depending on study quality and population characteristics. Larger prospective studies are required to confirm the findings of these observational studies.

## Figures and Tables

**Table 1:** Baseline Characteristics of Included Studies.

Author/Year	Setting (Country/Registry)	Study Design	Total Number of Participants	Mean/Median Age (Years)	PE/Outcome Defined As	Follow-Up Duration
Biere-Rafi 2013 [16]	Netherlands (PHARMO Record Linkage System)	Retrospective cohort study	3186 (acute PE 1998–2008)	(mean years ± SD) 61 ± 17	Recurrent pulmonary embolism confirmed by hospitalization records	Median 1529 days
Nguyen 2013 [17]	Denmark (Nationwide Cohort)	Nationwide cohort study	44,330 patients with VTE	(mean years ± SD) 62 ± 18 years	Hospitalized recurrent VTE (PE ± DVT)	Up to 13 years (1997–2009)
Schmidt 2014 [18]	Denmark (National Health Registries)	Combined nationwide cohort and nested case–control study.	27,862 with first-time VTE	N/A	Recurrent VTE confirmed by discharge registry	Up to 8.5 years (2004–2012 registry window)
Smith 2016 [19]	United States (Single health system)	Population-based inception cohort	2798 incident VTE	N/A	Recurrent VTE (PE or DVT) identified by ICD-9 codes	Up to 8 years (2002–2010 observation).
Brækkan 2017 [20]	Netherlands (MEGA follow-up study)	Prospective cohort study	2547 first VTE	Median 48 (IQR 37–58)	Recurrent VTE confirmed by medical record and imaging	Median 5.7 years
Hsu 2021 [21]	USA (Single-center hospital)	Retrospective single-center cohort	3097 patients with confirmed PE (522 [16.9%] on statins prior to admission)	(mean years ± SD) 69 ± 13	In-hospital mortality, short-term complications	During hospitalization
Stewart 2020 [22]	USA (Indiana statewide records)	Retrospective registry analysis	192,908 DVT or PE	Mean 67	Recurrent VTE via hospitalization data	Up to 13 years (2004–2017)
Wang 2021 [23]	China	Retrospective cohort study	365 patients with an ICD-confirmed diagnosis of pulmonary embolism (PE)	Median (IQR) 75.0 (66.0, 81.5)	Recurrent PE	Median 19.2 months (interquartile range: 10.6–26.2 months)
Siniscalchi 2022 [14]	International (RIETE Registry)	Prospective registry	31,169 acute PE	(mean years ± SD) 75 ± 11 74 ± 11 74 ± 11 Low, moderate and high intensity respectively	30-day all-cause mortality	30 days
Siniscalchi 2024 [15]	International (RIETE Registry)	Prospective registry	46,440 isolated DVT	(mean years ± SD) 72 ± 12	3-month mortality	3 months
Hagiya 2025 [24]	Global (WHO mortality database)	Epidemiological analysis	1,550,883	N/A	Global PE-related mortality	22 years (2001–2023)
Gressenberger 2025 [25]	Austria (Univ. Hospital Graz)	Retrospective data analysis	1590 acute PE	74 years [IQR, 66–80]	PE severity (ESC 2019 criteria)	30 days and 2 years

N/A: Not Available.

**Table 2:** Quality assessment of included studies using the Newcastle–Ottawa Scale (NOS).

Study (Author, Year)	Selection (0–4)	Comparability (0–2)	Outcome (0–3)	Total (0–9)	Quality
Biere-Rafi, 2013 [16]	★★★	★★	★★	7/9	Good
Nguyen, 2013 [17]	★★★★	★★	★★★	9/9	High
Schmidt, 2014 [18]	★★★★	★★	★★	8/9	Good
Smith, 2016 [19]	★★★★	★★	★★	8/9	Good
Brækkan, 2017 [20]	★★★	★★	★★	7/9	Good
Hsu, 2019 [21]	★★★	★★	★★	7/9	Good
Stewart, 2020 [22]	★★★	★	★★	6/9	Fair
Wang, 2021 [23]	★★★	★	★★	6/9	Fair
Siniscalchi, 2022 [14]	★★★	★	★★	6/9	Fair
Siniscalchi, 2024 [15]	★★★	★	★★	6/9	Fair
Gressenberger, 2025 [25]	★★★	★	★★	6/9	Fair
Global Trends, 2025 [24]	★★	★	★★	5/9	Fair (ecological, population-level, not patient data)

**Table 3:** Summary of Clinical Outcomes and Effect Estimates for Statin Use in Patients with PE or VTE.

Outcome	Study (Author, Year)	Effect Estimate (95% CI)	*p*-Value
Recurrent VTE (PE/DVT)	Biere-Rafi (2013) [16]	aHR 0.50 (0.36–0.70)	
	Nguyen (2013) [17]	HR 0.74 (0.68–0.79)	
	Schmidt (2014) [18]	aHR 0.72 (0.59–0.88)	
	Smith (2016) [19]	HR 0.74 (0.59–0.94)	
	Smith (2016, no-CVD) [19]	HR 0.62 (0.45–0.85)	
	Smith (2016, with CVD) [19]	HR 1.10 (0.70–1.70)	
	Brækkan (2017) [20]	HR 0.78 (0.46–1.31)	
	Stewart (2020) [22]	OR 0.66 (0.64–0.69)	
	Stewart (2020, PSM) [22]	OR 0.75 (0.72–0.79)	
	Wang (2021) [23]	OR 1.06 (0.52–2.09)	
	Wang (2021, PSM) [23]	OR 0.49 (0.19–1.26)	(*p* = 0.138)
	Siniscalchi (2022) * [14]	198 events—effect estimate not reported	
	Siniscalchi (2024) [15]	1.7% vs. 1.6%)	(*p* = 0.49)
Recurrent DVT	Schmidt (2014) [18]	aHR 0.64 (0.49–0.84)	
	Brækkan (2017) [20]	HR 0.66 (0.29–1.48)	
	Siniscalchi (2024) [15]	0.91% vs. 0.89%)	(*p* = 0.81)
	Hsu (2019) [21]	15% vs. 17% (NS)	
Recurrent PE	Biere-Rafi (2013) [16]	aHR 0.50 (0.36–0.70)	
	Schmidt (2014) [18]	aHR 0.82 (0.62–1.09)	
	Wang (2021) [23]	OR 1.06 (0.52–2.09)	
	Siniscalchi (2024) [15]	0.77% vs. 0.70%	(*p* = 0.005)
30-Day/Short-Term Mortality	Siniscalchi (2022) [14]	OR 0.65 (0.56–0.76)	*p* < 0.001
	Siniscalchi (2022, Fatal PE) [14]	OR 0.42 (0.28–0.62)	
	Siniscalchi (2022, Low statin) [14]	OR 0.51 (0.34–0.77)	
	Siniscalchi (2022, Moderate) [14]	OR 0.68 (0.57–0.81)	
	Siniscalchi (2022, High) [14]	OR 0.68 (0.51–0.92)	
	Gressenberger (2025) [25]	HR 0.85 (0.42–1.72)	
	Hsu (2019) [21]	OR 0.79 (0.49–1.27)	
	Siniscalchi (2024) [15]	aHR 0.77 (0.69–0.86)	
All-Cause/Long-Term Mortality	Biere-Rafi (2013) [16]	aHR 0.53 (0.41–0.69)	
	Hagiya (2025) [24]	3.49 → 2.42 deaths/100,000 (2001–2023)	

* Note: Effect estimates and *p*-values are presented as reported in the original publications. “Not reported” indicates data unavailable in the source studies (e.g., Siniscalchi et al., 2022 [14], which reported event counts only for recurrent VTE).

## Data Availability

All data generated or analyzed during this study are included in this article and its Supplementary Information files.

## Supplementary Materials

The following supporting information can be downloaded at: https://www.mdpi.com/article/10.3390/jcm14238333/s1, PRISMA 2020 Checklist.

## Abbreviations

The following abbreviations are used in this manuscript:

PE	Pulmonary Embolism
VTE	Venous Thromboembolism
DVT	Deep Vein Thrombosis
HMG-CoA	3-Hydroxy-3-Methylglutaryl-Coenzyme A
NOS	Newcastle–Ottawa Scale
PRISMA	Preferred Reporting Items for Systematic Reviews and Meta-Analyses
PROSPERO	International Prospective Register of Systematic Reviews
HR	Hazard Ratio
aHR	Adjusted Hazard Ratio
OR	Odds Ratio
IRR	Incidence Rate Ratio
CI	Confidence Interval
ICD	International Classification of Diseases
BMI	Body Mass Index
ESC	European Society of Cardiology
WHO	World Health Organization
RCT	Randomized Controlled Trial
RIETE	Registro Informatizado de la Enfermedad TromboEmbólica (Computerized Registry of Patients with Venous Thromboembolism)
HOPE-3	Heart Outcomes Prevention Evaluation-3 Trial
JUPITER	Justification for the Use of Statins in Prevention: an Intervention Trial Evaluating Rosuvastatin
CRD	Centre for Reviews and Dissemination (used in PROSPERO IDs, e.g., CRD420251166536)
CVD	Cardiovascular Disease
SD	Standard Deviation
IQR	Interquartile Range
PSM	Propensity Score Matching
ESC-2019	2019 European Society of Cardiology Guidelines for Pulmonary Embolism
Eur. Heart J.	European Heart Journal
Am. J. Med.	American Journal of Medicine
BMJ	British Medical Journal
N. Engl. J. Med.	New England Journal of Medicine
